# Microfluidic Systems to Study Neutrophil Forward and Reverse Migration

**DOI:** 10.3389/fimmu.2021.781535

**Published:** 2021-11-24

**Authors:** Kehinde Adebayo Babatunde, Jose M. Ayuso, Sheena C. Kerr, Anna Huttenlocher, David J. Beebe

**Affiliations:** ^1^ Department of Pathology & Laboratory Medicine, University of Wisconsin, Madison, WI, United States; ^2^ Carbone Cancer Center, University of Wisconsin, Madison, WI, United States; ^3^ Departments of Pediatrics and Medical Microbiology and Immunology, University of Wisconsin-Madison, Madison, WI, United States; ^4^ Department of Biomedical Engineering, University of Wisconsin, Madison, WI, United States

**Keywords:** neutrophils, microfluidic, reverse migration, forward migration, migration

## Abstract

During infection, neutrophils are the most abundantly recruited innate immune cells at sites of infection, playing critical roles in the elimination of local infection and healing of the injury. Neutrophils are considered to be short-lived effector cells that undergo cell death at infection sites and in damaged tissues. However, recent *in vitro* and *in vivo* evidence suggests that neutrophil behavior is more complex and that they can migrate away from the inflammatory site back into the vasculature following the resolution of inflammation. Microfluidic devices have contributed to an improved understanding of the interaction and behavior of neutrophils *ex vivo* in 2D and 3D microenvironments. The role of reverse migration and its contribution to the resolution of inflammation remains unclear. In this review, we will provide a summary of the current applications of microfluidic devices to investigate neutrophil behavior and interactions with other immune cells with a focus on forward and reverse migration in neutrophils.

## Introduction

Neutrophils are the most abundant white blood cell, with 1 × 10^11^ new cells produced in the bone marrow daily with a short lifespan of about ∼7–24 hours in the blood (1). Neutrophils, also known as polymorphonuclear cells (PMNs) are terminally differentiated leukocytes with typical lobulated nuclei and antimicrobial cytoplasmic granules. Neutrophils are essential immune cells, crucial for the body’s innate immune response, and are often the first line of defense against invading pathogens (2). Neutrophils are involved in various disease processes, including pathogen infection (3), pulmonary diseases (4), cardiovascular diseases (5), inflammatory disorders (6) and cancer (7). Importantly, modifying or reducing neutrophil influx by pharmacological treatment can serve as a potential therapy. For example, during infection, enhancing neutrophil numbers and function by G-CSF treatment has been shown to be beneficial to treat severe neutropenia, particularly in the case of chemotherapy-induced neutropenia (8, 9). Furthermore, in pulmonary diseases and atherosclerosis, inhibition of neutrophil functions such as blocking neutrophil recruitment and targeting NETs has been demonstrated to be helpful (10–12). Neutrophils are challenging to study because they are short-lived effector cells of the innate immune system with a complex and diverse role in fighting pathogens, acute inflammation and they actively participate in several diseases including cancer (13).

Reverse migration has been described as a process whereby neutrophils migrate away from sites of tissue damage or infection once the infection has been resolved (14). The resolution of inflammation is generally thought to be driven by macrophages by efferocytosis of dead neutrophils at the wound site (15). However, following the identification of retrotaxis as an alternative path to resolution of neutrophilic inflammation in zebrafish (14), the functional role of neutrophil reverse migration during an immune response is still unclear as it is challenging to study in a human model. Evidence that suggests that neutrophil reverse migration can be a protective response, but it can also be detrimental and lead to systemic inflammation (16, 17). The protective response is attained by facilitating an efficient immune response resolution once the infection has been cleared, and this has been demonstrated using both mouse and zebrafish models (18, 19). Animal models have provided strong evidence of reverse migration in neutrophils and its impact on disease, but these findings do not always translate to human neutrophil activity.

Microfluidic approaches represent an integrative technology that enables customizable studies of primary leukocytes *ex vivo*. Microfluidic devices have contributed to improved understanding of the interaction and behavior of neutrophils *ex vivo* in 2D and 3D microenvironments. To better understand forward and reverse migration in neutrophils and the regulatory interactions between neutrophils and other cells, factors and molecular mechanisms driving this process, there is a need to identify appropriate *in vitro* models to study this process with human cells. In addition, these *in vitro* or *ex vivo* systems should account for relevant geometries, cell-cell interactions, and cell-matrix interactions. This review will provide a summary of the current applications of microfluidic devices to investigate neutrophil behavior and interactions with other immune cells with a focus on forward and reverse migration in neutrophils. The current state of knowledge regarding the mechanisms that control reverse migration are also reviewed and the potential for microfluidic devices to continue to help unravel the underlying biology that drives these phenomena is described. Finally, the use of microfluidic devices to improve our understanding of neutrophil behavior in other contexts (e.g. cancer, pathogen interaction) is discussed.

### Neutrophil Functions

Neutrophils are phagocytes that internalize foreign pathogens into phagolysosomes *via* receptor‐mediated phagocytosis. They contain antimicrobial granules filled with multiple enzymes such as cathepsins, elastases, and myeloperoxidases (20). These enzymes are responsible for digesting internalized pathogens in the phagolysosomes in a process called degranulation (20). Neutrophils have several complex pathogen-killing mechanisms that include the formation of neutrophil extracellular traps (NETs), and reactive oxygen species (ROS) generation (21). Neutrophils produce ROS *via* NADPH oxidase to kill both extracellular and intracellular pathogens, while NETs consist of decondensed chromatin fibers laced with granular proteins and histones, which are deployed to immobilize and kill invading pathogens (22). Neutrophils are migratory cells, and upon sensing infection, they are the first cells to migrate to the infection site (17). To traffic toward infection sites, neutrophils respond to chemokine gradients and then interact with endothelial vessels undergoing rolling and adhesion, before extravasation, and finally, migration (23). These processes have been extensively described and reviewed (17, 24). However, neutrophils can also migrate away from the infection site, once the infection has been resolved in a newly described reverse migration process (14, 25, 26).

## Reverse Migration in Neutrophils

Neutrophils act as first responders during an innate immune response and are often the first cells to arrive at a site of infection or injury. After neutrophils execute their antimicrobial function at the site of infection, timely clearance of neutrophils is crucial to maintain homeostasis (27, 28). Macrophages carry out clearance of dead neutrophils at the site of infection *via* apoptosis or necrosis and subsequent phagocytosis (17), however recent evidence suggests that live neutrophils recirculate away from the site of infection and back into the blood vessels. Reverse migration in neutrophils was first suggested by Hughes et al. The authors used a rat model that describes the tracking and migration of radiolabeled neutrophils from an inflamed glomerular capillary back to the main circulation (29).

The mechanism involved in reverse migration and its visualization *in vivo* was reported by Mathias et al. The authors demonstrated that most neutrophils at the site of tissue damage reverse migrated away from the wound back to blood circulation (14). In agreement with Mathias et al. study, Buckley and colleagues reported that human neutrophils undergo reverse transendothelial migration (rTEM) through a tumor necrosis factor-α (TNF-α)-activated endothelial monolayer (25). Interestingly, Tauzin and colleagues also showed that macrophages could drive reverse migration in neutrophils using the zebrafish model. The authors found that reverse migration of neutrophils from a wound site was mediated by ROS-Src family kinase signaling in macrophages, providing a mechanism by which macrophages modulate resolution of neutrophil-mediated inflammation (30, 31).

Multiple phenotypic markers of reverse migrated neutrophils have been identified. Neutrophils that undergo reverse migration have a high expression level of intercellular adhesion molecule-1 (ICAM-1^high^) and a low expression level of C-X-C motif chemokine receptor 1 (CXCR1^low^) compared to neutrophils in blood circulation which are ICAM-1^low^ and CXCR1^high^ (32, 33). Interestingly, ICAM-1^high^/CXCR1^low^ neutrophils were found to be increased in patients with systemic inflammation (25). Wang and colleagues visualized and imaged neutrophils that reverse migrated from an inflammatory site in the liver to the lungs and bone marrow (34). While these studies (**Table 1**) indicated that neutrophils reverse migrate, the fate of reverse-migrated neutrophils and the underlying mechanism of reverse migration remains unclear.

**Table 1:** Summary of studies on reverse migration.

Title	Model	Major finding	Ref
**Reverse migration**			
Resolution of inflammation by retrograde chemotaxis of neutrophils in transgenic zebrafish.	*In vivo*: Zebrafish	Neutrophils can display directed retrograde chemotaxis back toward the vasculature	(14)
Neutrophil migration in infection and wound repair: going forward in reverse.	*In vivo* and *In vitro*	Review article	(17)
Identification of a phenotypically and functionally distinct population of long-lived neutrophils in a model of reverse endothelial migration.	*In vitro*	Neutrophils can migrate in a retrograde direction across endothelial cells	(25)
Neutrophil integrin affinity regulation in adhesion, migration, and bacterial clearance.		Review article	(27)
Neutrophil Metabolic Shift during their Lifecycle: Impact on their Survival and Activation.		Review article	(27)
Getting to the site of inflammation: the leukocyte adhesion cascade updated.		Review article	(29)
Redox and Src family kinase signaling control leukocyte wound attraction and neutrophil reverse migration.	*In vivo*: Zebrafish	Neutrophil-macrophages interaction induce resolution *via* neutrophil reverse migration	(30)
Neutrophils in the Tumor Microenvironment.		Review article	(33)
Visualizing the function and fate of neutrophils in sterile injury and repair.	*In vivo*: Mouse	Neutrophils can migrate back into the circulation as a physiological process and return to the lung, potentially to be deactivated or reprogrammed	(33)

**Figure 1:**
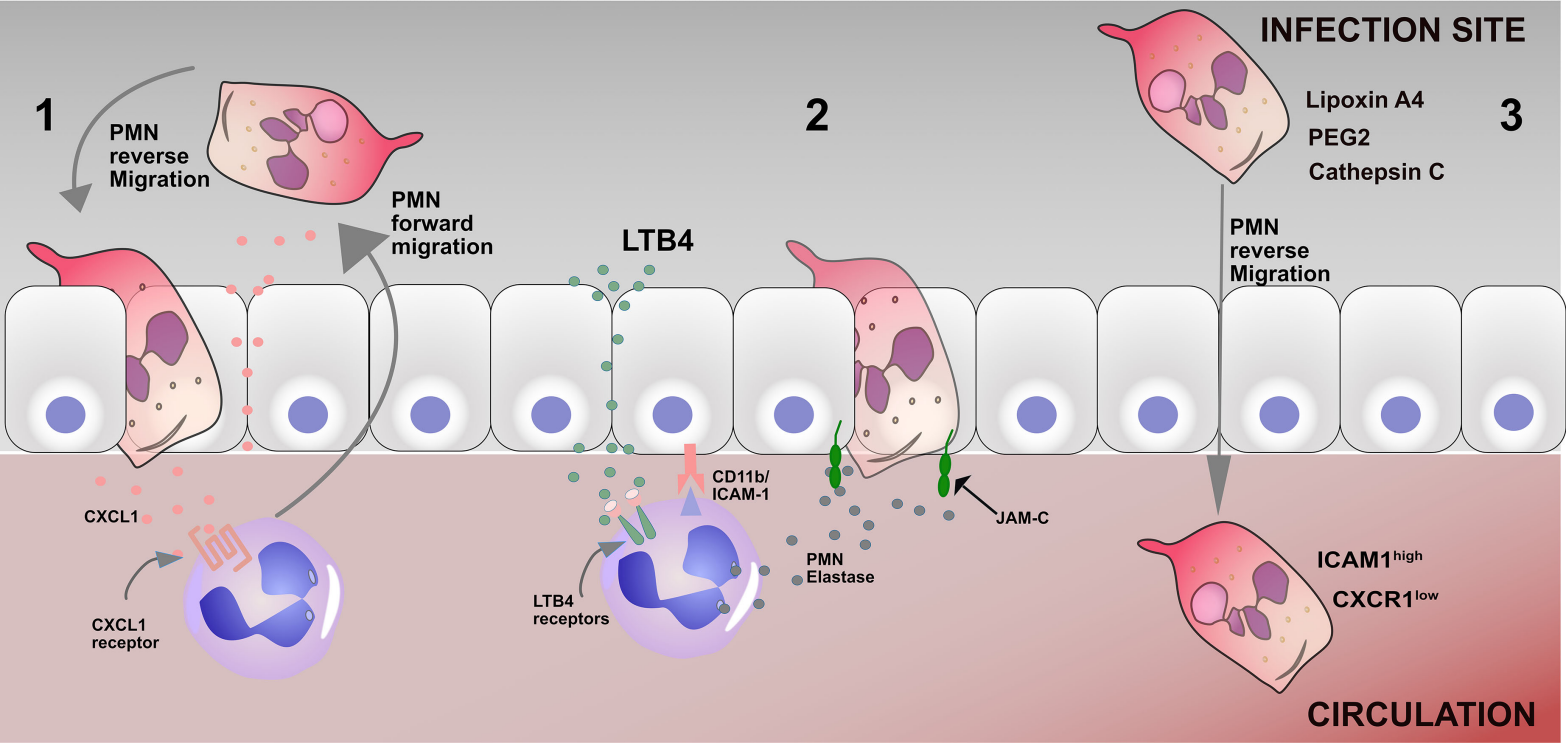
Mechanisms of reverse migration in neutrophils 1. Leakage of CXCL1 into the endothelium as a result of a breached endothelium, therefore driving neutrophils to reenter the circulation; 2 Leukotriene B4 induces neutrophil elastase release (NE), which in turn cleaves endothelial JAM-C and subsequently results in the disruption of the endothelial junction and promotes neutrophil reverse migration. 3 Many factors, including Lipoxin A4, PEG2, and cathepsin C, can promote neutrophil reverse migration. The phenotype of reverse migrated neutrophils are ICAM1^high^ CXCR1^low^.

The mechanisms that mediate reverse migration in neutrophils from sterile injury remain unclear. However, factors like chemotactic repellents, neutrophil-endothelial interaction, and chemokine receptors have been identified as contributing to reverse migration in neutrophils (35) (**Figure 1**). Neutrophils migrate from the circulation to the infection site by breaching the endothelium (36). Compromise in the structural and functional integrity of the endothelial junction and permeability could play a crucial role in the mechanism of reverse migration in neutrophils (37). During inflammation, structural damage occurs at endothelial junctions, thus increasing endothelial permeability. There can also be diffusion of chemokines from damage sites that influence vascular permeability. It is possible that these factors may affect neutrophil reverse migration and transmigration across vessel walls (38, 39).

Junctional adhesion molecules are members of an immunoglobulin subfamily, consisting of JAM-A, -B, -C, -4, endothelial cell selective adhesion molecule (ESAM), and coxsackievirus and adenovirus receptor (CAR) that are specifically enriched at the tight junctions of cell-cell contacts (40). Woodfin et al. demonstrated that neutrophil reverse transmigration is regulated by the junctional adhesion molecule pathway in mouse models (26). Accordingly, inhibiting the junctional adhesion molecule pathway reduces reverse migration in neutrophils in septic mice (41). Furthermore, Colom and colleagues reported that CD11b and ICAM-1 interaction lead to junctional adhesion molecule cleavage by neutrophil elastase, promoting reverse transmigration in neutrophils (42) (**Figure 1**). The study further showed that the leukotriene B-neutrophil elastase pathway may contribute to neutrophil reverse transmigration in mice (42). Li et al. demonstrated that blocking LTB_4_ receptors could decrease reverse transmigration of neutrophils in mice (43). In another study by Hirano and colleagues, it was demonstrated that inhibition of JAM-C degradation significantly decreases neutrophil reverse transmigration in septic mice (41).

Studies have begun to identify some chemokines and chemokine receptors that are involved in neutrophil reverse migration. Chemokine receptors such as CXCR1 and CXCR2 are crucial for neutrophils to sense chemokines during cell migration. Interestingly, a study showed that reverse migrated neutrophils had decreased expression of CXCR1 (25). Therefore, it was speculated that those neutrophils might lose their ability to sense chemokine cues and migrate in a reverse direction. However, the actual evidence and mechanism remain unclear. Another study using zebrafish showed that CXCL8a and CXCR2 (human equivalent of CXCR1) are required for neutrophils to undergo reverse migration. The authors further showed that a CXCR2 knockout in zebrafish had impaired reverse migration of neutrophils (44).

Wang and colleagues, in their study, used advanced intravital imaging techniques in mice to visualize neutrophil dynamics within a thermal hepatic injury site. The authors reported that neutrophils migrate away from the injury site following tissue repair back into the vasculature. They further demonstrated that the reversed migrating neutrophils traffic to the lung and eventually to the bone marrow, where they die *via* apoptosis (34). Some studies have molecular mechanisms that regulate the reverse migration in neutrophils. For example, Elk and colleagues showed the role of hypoxia-inducible factor 1 subunit alpha (HIF1α) in suppressing reverse migration of neutrophils in zebrafish (45). The authors also noted that the activation of HIF1α reduced reverse migration of neutrophils and delayed inflammation resolution in zebrafish. A more recent study showed that the paracrine factor myeloid derived growth factor (MYDGF) affected neutrophil reverse migration through a HIF1a dependent pathway (46).

Chemotactic repellency in the inflammatory site is another mechanistic hypothesis for reverse migration in neutrophils (17). CXCL8 is a potent neutrophil chemoattractant in human inflammation (47). CXCL8 binds to the G protein-coupled receptors CXCR1 and CXCR2 (48). Tharp et al. reported that CXCL8 functions as a chemoattractant and chemorepellent in lower and higher concentrations, respectively. CXCL8 as a chemorepellent has been shown to drive reverse migration in neutrophils (49). Other studies have reported eicosanoid prostaglandin E_2_ (PGE2), and macrophages as chemorepellents that drive reverse migration in zebrafish neutrophils (50). These studies (**Table 2**) collectively showed the complexity and diversity of the suggested mechanisms driving reverse migration in neutrophils. Thus, we need to improve our knowledge about neutrophil reverse migration and its effect on immune response and inflammation.

**Table 2:** Summary of studies on the mechanism of reverse migration in neutrophils.

Title	Model	Major finding	Ref.
**Mechanism of neutrophil reverse migration**			
Neutrophil migration in infection and wound repair: going forward in reverse.		Review article	(21)
Identification of a phenotypically and functionally distinct population of long-lived neutrophils in a model of reverse endothelial migration.	*In vitro*	Neutrophils can migrate in a retrograde direction across endothelial cells	(24)
The junctional adhesion molecule JAM-C regulates polarized transendothelial migration of neutrophils *in vivo*.	*In vivo*: Mouse	Neutrophils exhibit transendothelial migration *via* the junctional adhesion molecule.	(25)
Visualizing the function and fate of neutrophils in sterile injury and repair.	*In vivo*: Mouse	Neutrophils can migrate back into the circulation as a physiological process and return to the lung, potentially to be deactivated or reprogrammed	(33)
Big insights from small volumes: deciphering complex leukocyte behaviors using microfluidics.	*Ex vivo*: Microfluidics	Review article	(34)
Leukocyte migration in the interstitial space of non-lymphoid organs.		Review article	(35)
Local microvascular leakage promotes trafficking of activated neutrophils to remote organs.	*In vivo*: Mouse	Increase in microvascular leakage induces reverse migration in neutrophils.	(36)
Leaking chemokines confuse neutrophils.		Review article	(38)
JAM-C regulates unidirectional monocyte transendothelial migration in inflammation.	*In vivo*: Mouse	Blockade of JAM-B/-C interaction reduced monocyte numbers in the extravascular compartment *via* reverse transmigration.	(39)
Leukotriene B4-Neutrophil Elastase Axis Drives Neutrophil Reverse Transendothelial Cell Migration *in vivo.*	*In vivo*: Mouse	LTB_4_-neutrophil elastase pathway can promote reverse transendothelial migration in neutrophils	(41)
Substance P-regulated leukotriene B4 production promotes acute pancreatitis-associated lung injury through neutrophil reverse migration.	*In vivo*: Mouse	Substance P regulates the production of LTB_4_ *via* PKCα/MAPK pathway.	(42)
Chemokine Signaling and the Regulation of Bidirectional Leukocyte Migration in Interstitial Tissues.	*In vivo*: Zebrafish	CXCL-8/CXCR-2 as a specific ligand-receptor pair orchestrates neutrophil chemokinesis in interstitial tissues during neutrophil reverse migration and resolution of inflammation	(43)
Activation of hypoxia-inducible factor-1alpha (HIF-1alpha) delays inflammation resolution by reducing neutrophil apoptosis and reverse migration in a zebrafish inflammation model.	*In vivo*: Zebrafish	Hypoxia-inducible factor-1alpha activated neutrophils exhibited reduced reverse migration.	(44)
Myeloid-derived growth factor regulates neutrophil motility in interstitial tissue damage.	*In vivo*: Zebrafish	Myeloid-derived growth factor mutant neutrophils exhibited impaired reverse migration.	(45)
Neutrophil chemorepulsion in defined interleukin-8 gradients *in vitro* and *in vivo*.	*Ex vivo*: Microfluidic	Neutrophil undergo chemo-repulsion in response to IL-8 gradient.	(48)

What is the final fate of reverse migrated neutrophils? Several studies have suggested several outcomes. For example, a study showed that neutrophils could be found in circulation for at least 48 hours after migrating away from the wound site using the zebrafish model (51). Imbalance in reverse migration could lead to multiple organ failure (52). For example, neutrophils expressing ICAM-1^high^/CXCR1^low^ markers were found in the circulation of patients with acute pancreatitis that developed acute lung injury (53). The same authors reported a similar observation in a mouse model of acute pancreatitis that an increased level of neutrophils expressing ICAM-1^high^/CXCR1^low^ markers were found in circulation (53). Another study showed that following ischemia–reperfusion injury in mice, ICAM-1^high^/CXCR1^low^ neutrophils were found to be resident in the lungs (26). Colom and colleagues, in agreement, showed that experimentally induced rTEM resulted in increased organ damage in the lungs, liver, and heart (42). Furthermore, neutrophils have also been demonstrated to migrate away from the site of infection and re-localize to the lymph nodes (54) or bone marrow (55) to affect host defense. The clinical implication of this re-localization is that neutrophils can shuttle live pathogens to lymph nodes (56) (**Table 3**).

**Table 3:** Summary of studies on the fate of reverse migrated neutrophils.

Title	Model	Major finding	Ref.
**Implication and fate of reverse-migrated neutrophils**			
Spatiotemporal photolabeling of neutrophil trafficking during inflammation in live zebrafish	*In vivo:* Zebrafish	Visualization of the origin and fate of neutrophils during induction and resolution of inflammation	(50)
Reverse-migrated neutrophils regulated by JAM-C are involved in acute pancreatitis-associated lung injury.	*In vivo*: Mouse	Neutrophils that are recruited to the pancreas may reverse migrate back into circulation and could potentially contribute to acute lung injury during acute pancreatitis	(52)
Microbe-dependent lymphatic migration of neutrophils modulates lymphocyte proliferation in lymph nodes.	*In vivo*: Mouse	Skin-egressing neutrophils migrate to the lymph nodes to augment lymphocyte proliferation in draining lymph nodes.	(53)
Neutrophils rapidly migrate *via* lymphatics after Mycobacterium bovis BCG intradermal vaccination and shuttle live bacilli to the draining lymph nodes	*In vivo*: Mouse	Neutrophils migrate to lymphoid tissue and can shuttle live microorganisms.	(55)

The functional role of reverse migration in neutrophils during inflammation needs further studies. However, current evidence suggests that it could be context dependent. Reverse migration in neutrophils can be both a protective response by facilitating an efficient resolution to an innate immune reaction and also a tissue-damaging event (17, 34, 42, 57, 58). Migration of activated neutrophils away from the infection sites through reverse migration may resolve an inflammatory reaction. However, reverse migrating neutrophils re-entering the vasculature can spread infections (26, 59). Few studies have shown strong *in vivo* evidence that neutrophils can reverse migrate into circulation (26, 42). For example, Elks et al. and Tharp et al. demonstrated that reverse migration in neutrophils could promote inflammation resolution using a zebrafish model. These studies also showed that inhibition of reverse migration in neutrophils by macrophage depletion and activation of HIF1α resulted in more wound damage due to infiltration of neutrophils (45, 49).

Pathologically, several studies have shown that reverse migrated neutrophils contribute to the spreading of infection and distant organ dysfunction. For example, Colom et al. and Woodfin et al. demonstrated that inhibition of reverse migration in neutrophils protected the host from distant organ damage using a murine model of ischemia-reperfusion (26, 42). Though the underlying mechanism of how reverse migrated neutrophils drive distant organ damage remains unclear, they speculate that the mechanism could result from cellular interaction between reverse migrated neutrophils and circulating cells. In a recent study by Bernut et al. the authors demonstrated that the loss of cystic fibrosis transmembrane conductance regulator (CFTR) delays resolution of inflammation by reducing neutrophil reverse migration in the context of sterile inflammation using a zebrafish model of cystic fibrosis (CF). The authors further reported that the pathogenic mechanisms leading to persistent neutrophilic inflammation in CF involve CFTR-related defect in reverse migration of neutrophils (16).

Another study by Hirano and colleagues showed that neutrophils with a similar phenotype to reverse migrated neutrophils modulate T cell functions by suppressing their proliferation in a CD18/CD11b dependent manner (53). Furthermore, inflammatory conditions that drive reverse migration in neutrophils are linked with a high expression level of ICAM and ROS generating neutrophils in the pulmonary vasculature, a response that is also associated with lung inflammation (26). Interestingly, in chronic inflammatory conditions like atherosclerosis and rheumatoid arthritis, there has been a report of high numbers of ICAM-1^high^ neutrophils in circulation (25), suggesting that reverse migrated neutrophils may be associated with the pathogenesis of these inflammatory diseases. In conclusion, most reverse migration studies have been demonstrated using animal models. However, studies of reverse migration in humans are limited and can be challenging to investigate. Thus, the use of microfluidic platforms to study reverse migration in human neutrophils could be helpful.

### Microfluidic Devices for Studying Reverse Migration of Neutrophils

The migration of neutrophils is driven by sensing and responding to chemokines such as hydrogen peroxide (H_2_O_2_) (60, 61), interleukin 8 (IL-8) (62), macrophage-inflammatory protein-2 (MIP-2) (63), keratinocyte-derived chemokine (KC) (63), and pathogen-derived chemokines such as N-formyl-methionyl-leucyl-phenylalanine (*f*MLP) (64), and the complement system (C5a) (65). After migration, neutrophils release pro-inflammatory cytokines, proteolytic enzymes, and reactive oxygen species (ROS) to eliminate bacteria and pathogens. After neutrophils execute their antimicrobial function at the site of infection, timely clearance of neutrophils is crucial to maintain homeostasis (27, 28). A delicate balance between neutrophil migration into the tissues and their subsequent removal from the infection site must be achieved and it seems increasingly likely that reverse migration plays an important role in the resolution of inflammation. In addition, this balance is also maintained by efferocytosis of dead neutrophils by macrophages at the site of infection.

Reverse migration ensures efficient clearance of neutrophils as soon as the infection is cleared. Neutrophils home to the bone marrow after reverse migration where they are finally cleared. Neutrophil reverse migration will facilitate removing excessive neutrophils from the inflamed tissues in addition to the conventional mechanism of neutrophil removal from tissues by apoptosis, followed by efferocytosis by macrophages. In comparison to several studies on the forward migration of neutrophils, little is known about the mechanism of reverse migration of neutrophils. A few studies have demonstrated reverse migration in neutrophils using animal models, primarily mice and zebrafish (18, 19). Though these models have allowed researchers to understand the behavior of neutrophils in a complex environment and physiologically relevant systems, they are expensive to set up, have low throughput, variability is high, and do not always translate to human neutrophil activity. More so, studying neutrophil interaction with other immune cells during an immune response using these models can be challenging because of their complexity. There is a need to develop novel experimental platforms for studying and monitoring reverse migration in neutrophils that mimic the physiological microenvironment of *in vivo* systems and permit the study of human neutrophils.

Microfluidic devices are useful to study the immune response because of their customizable geometries, allowing researchers to recreate complex *in vivo* structures such as blood vessels, epithelial barriers, and organized cultures that include multiple cell types. These factors and requirements make them appropriate for studying the behaviors, interactions, and control of the spatiotemporal presentation of signaling molecules in primary human cells. They can be designed to incorporate crucial components like cells, tissues, and the biological architectures crucial for modeling the human immune system. Furthermore, microfluidic devices have been designed to study and monitor the functions of immune cells in real-time and at a single-cell resolution (66).

**Figure 2:**
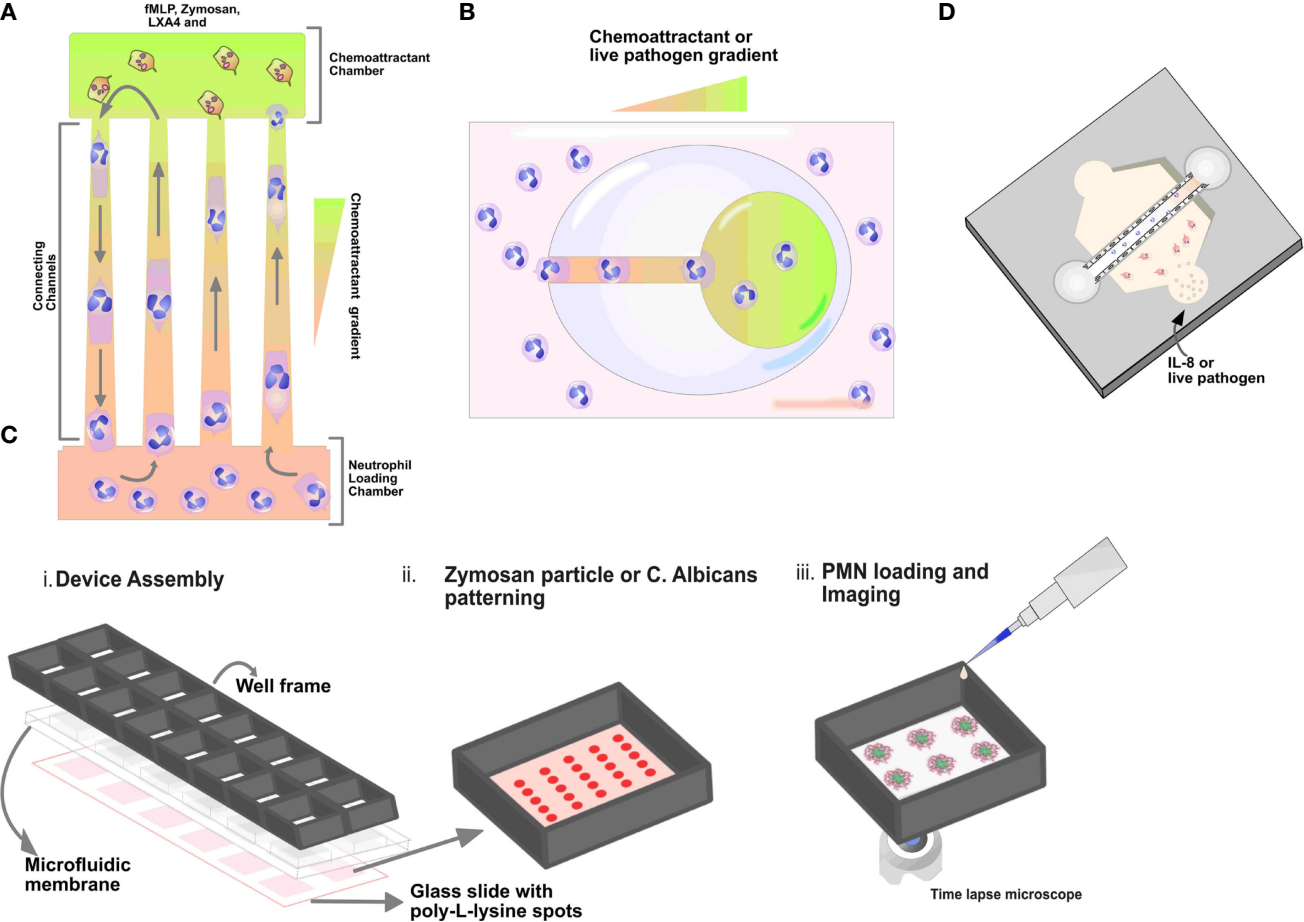
Schematics of microfluidic devices. **(A)** Tapered channel showing neutrophils migrating from the cell loading chamber (bottom) to the chambers loaded with chemoattractant fMLP (top). **(B)** Schematic illustration of egg-shaped chip. It consists of an inner chemoattractant micro-chamber and a large egg-shaped chamber connected by a straight channel. Neutrophils are seen migrating towards fMLP gradient in the egg chip shaped device. **(C)** Micropatterned *C. albicans* or zymosan particles (red spots) array for the quantification of neutrophil swarming i) Schematic illustrations showing the assembly of the 16 well open chamber device, ii) zoom‐in of one of the zymosan particle patterning in the wells and iii) subsequent neutrophil cells loading and imaging **(D)** Organotypic microfluidic devices include a model vasculature containing endothelial cells in a relevant lumen geometry. These devices use both chemokines and live pathogens to induce migration.

Several research groups have designed and developed microfluidic devices to study neutrophil behavior and function in real-time. For example, several studies have demonstrated neutrophil migration (67, 68), NETosis (69, 70) and ROS generation (71, 72) using microfluidic devices (**Figure 2**). Furthermore, several microfluidic devices have been developed to investigate neutrophil interaction with live pathogens. For example, a study by Hopke et al. designed a device containing an array of fungal clusters to observe and monitor coordinated migration in human neutrophils (73). In addition to fungal pathogens, microfluidic devices have been designed to monitor and investigate neutrophil-bacteria interactions (74). These microfluidic models have allowed researchers to understand key components of the neutrophil response in a highly controlled micro-environment (**Figure 2**).

Given the numerous microfluidic applications to study neutrophil behavior, researchers are leveraging microfluidic devices to study and understand the mechanisms involved in neutrophil reverse migration. For example, Tharp and colleagues first described the concept of reverse migration in neutrophils against a chemokine gradient using a microfluidic migration assay (49). The authors used a microfabricated microfluidic linear-gradient device. They reported that human neutrophils exhibited persistent and directional migration away from the IL-8 chemokine gradient. The authors described this behavior as chemorepulsion or fugetaxis and demonstrated that fugetaxis is dependent on phosphoinositide-3-kinase, RhoGTPases, and associated proteins. Furthermore, they showed that the disruption signaling molecules like Cyclic Adenosine Monophosphate (cAMP) and the activity of Protein Kinase C isoforms could revert fugetaxis to a chemoattractant response (49).

Another study by Hamza et al. designed a microfluidic device to describe retrotaxis in neutrophils and further identify the experimental conditions that drive this migration pattern. The authors reported that the microfluidic device comprises a main loading channel and an inverted U-shaped migration channel. The microdevice mimics *in vivo* mechanical constraints encountered by neutrophils during migration. A chemoattractant gradient is established through diffusion between the side channels and the main channel and the highest concentration is established in the U region of the device. In the study, *f*MLP or/and Lipoxin A4 (LXA_4_) and Alexa-Fluor-488-labelled zymosan were used as chemoattractants (**Figure 2A**). Following the initial migration of neutrophils, the authors observed that the majority of the neutrophils (90%) exhibited reverse migration in the presence of lipoxin A4 (LXA_4_), a well-established mediator of inflammation resolution compared to other chemoattractants. They also reported that retrotaxis stops after neutrophils encounter a target, which they can phagocytose (75). In summary, this study demonstrated that lipoxin A4 could induce reverse migration in neutrophils compared to *f*MLP and zymosan using a microfluidic platform.

In another study by Wang et al., the authors designed a microfluidic device to study the effect of mechanical confinement on the migration patterns of neutrophils toward chemokine gradients. The microfluidic device consists of an array of tapered channels. The tapered channels connect one shared cell-loading channel to multiple chemoattractant chambers. A chemoattractant gradient is established along the migration channels to monitor and track migration, from the cell loading chamber to the chemoattractant chambers (**Figure 2A**). In the study, the authors used fMLP, LTB_4_, and IL-8 as chemoattractants. The authors identified and described four migration patterns, including reverse migration. Notably, the authors reported that a higher percentage of neutrophils exhibited reverse migration in the IL-8 gradient (76). There are few studies showing the use of microfluidic systems to investigate the mechanism of reverse migration in neutrophils. These mechanisms include chemokine receptor desensitization (77) and chemokinetic *versus* chemotactic responses (78).

Current proposed experimental approaches to study inflammation resolution by reverse migration in neutrophils suggest that the dynamics of neutrophil migration into and out of the infection site might be regulated by the balance between the killing of microbe-like particles and retrotaxis (79). However, studies showing that lipid mediators could improve neutrophil migration away from infection sites suggest that reverse migration in neutrophils could be modulated by several intercellular signals (58, 75). Furthermore, macrophages may have a role in driving reverse migration in neutrophils (17, 57). As such, studies investigating the interaction of neutrophils and macrophages using a more advanced microfluidic device could improve our understanding of reverse migration in neutrophils in the context of inflammation and infection. Thus, there is an increasing need for alternative platforms that allow us to study reverse migration in human neutrophils. In this context, microfluidic devices that model the relevant physiological micro-environment during inflammation *ex vivo* present a promising tool to achieve this goal.

## Microfluidic Devices for Studying Neutrophil Biology

### Microfluidic Devices for Neutrophil-Chemokine Interaction

Microfluidic devices have been used to study neutrophil behavior *ex vivo* using known chemokines to model an infectious source (80). The use of well-established/known chemoattractants like fMLP, IL-8, LTB_4_, and C5a allows researchers to study and observe the behavior and migration of neutrophils toward the chemokine gradient. For example, researchers have described two-dimensional microfluidic models for studying migration in neutrophils towards various chemokines (80). The two-dimensional model consists of an array of tapered channels that connect the cell-loading chamber to several chemoattractant chambers (**Figure 2A**). Migration in neutrophils in the device is monitored by establishing a chemoattractant gradient across the tapered channel (76). In addition, the device was used to compare chemotaxis in human leukemia (HL-60) differentiated neutrophil-like cells to primary neutrophils (80). In another study by Boribong et al., the authors designed a 2D microfluidic device to demonstrate decision making in primary neutrophils between two chemoattractants: an end target chemoattractant (e.g., bacterial infection, fMLP) vs. an intermediary chemoattractant (e.g., LTB_4_, inflammatory signal). The authors reported that naive neutrophils migrate toward the primary end target signal in higher percentages than the secondary intermediary signal (68). Understanding these decision-making mechanisms in neutrophils may lead to the development and formulation of therapies that reduce neutrophil off-target organ damage (68).

#### Microfluidic Devices for Neutrophil-Live Pathogens Interaction

Pathogens like bacteria and fungi release several inflammatory signals and factors that drive neutrophil response at the infection site. These signals include pathogen-associated molecular patterns (PAMPs) and pathogen-derived peptides. When these signals are released, they activate the endothelial cells lining blood vessels, which initiates neutrophil extravasation. Neutrophils are equipped with several tools designed to kill pathogenic and non-pathogenic infections like phagocytosis, swarming, ROS generation, and NETosis. Several studies have reported the interaction of neutrophils and bacterium either using *in vitro* model or a 3D microfluidic model. For example, Moreland and colleagues showed that primary neutrophils displayed more chemotactic response to live bacteria than bacteria-related peptides like liposaccharide (LPS). The authors also reported that neutrophils exhibited more migratory ability when challenged with live *Escherichia coli* than LPS (81).

Microfluidic devices have also been used to investigate the interaction of neutrophils and live bacteria. Ellett and colleagues reported using a 2D microfluidic device, also known as the egg chip. The microfluidic egg chip consists of a large egg-shaped chamber with a single entrance channel connected to the inner central reservoir. A chemoattractant gradient is established in the device from the outer larger egg-shaped chamber through the connecting channel to the inner chemoattractant chambers. The device allows the researcher to monitor and analyze the interaction between isolated neutrophils and *Staphylococcus aureus*, including neutrophil recruitment and killing ability (74) (**Figure 2B**). A more advanced and physiologically relevant microfluidic model has recently been reported to study neutrophils and bacteria interaction. In a study by Hind et al., they demonstrated neutrophil-*Pseudomonas aeruginosa* interaction using an organotypic lumen seeded with endothelial cells to form a microvessel. The organotypic lumen was formed by molding a hollow structure in a hydrogel that is then seeded with endothelial cells to form a luminal monolayer. In this study, the authors reported that neutrophil activation and the response was increased due to endothelial secreted IL-6 and GM-CSF (23). The authors also reported that the presence of endothelial cells lining the lumen surface increases neutrophil life span.

The interaction of neutrophils and fungi using the microfluidic device has also been investigated, especially *Aspergillus fumigatus*. *Aspergillus fumigatus* is an opportunistic fungal pathogen that mainly affects immunocompromised and neutropenic patients. It is difficult to treat and results in high mortality in these patient populations (82). For example, a study by Jones et al. leveraged a microfluidic device consisting of a well array filled with a uniform concentration of chemoattractant. The design allowed a close interaction between the neutrophils and *A. fumigatus* conidia. In a subsequent study, neutrophils were required to migrate along a chemoattractant gradient to reach chemotaxis chambers loaded with *A. Fumigatus*. These design settings replicate chemoattractant gradients like LTB_4_ and *f*MLP that are established in tissues during inflammation and helps unravel the importance of directed neutrophil migration before interactions with *A. Fumigatus* (83). The authors demonstrated that primed neutrophils exhibit migratory responses toward *A. fumigatus* conidia and ultimately inhibited its growth. Interestingly, the inhibition of fungal hyphal growth is counteracted by the fungus *via de novo* tip formation and the growth of new hyphae near the neutrophil-fungi interaction site. This observed fungal behavior was found to be NADPH oxidase and NETosis independent using a microfluidic device (82).

Apart from studying the migratory ability in neutrophils toward fungal pathogens; swarming, a complex coordinated migration has also been described using a micro-patterned swarming device. Briefly, the authors designed a glass swarming assay by micro-patterning live *Candida albicans* on glass slides to serve as targets for neutrophils swarms. For neutrophil- *Candida albicans* interaction and incubation, the micropatterned glass slides were placed in a commercially available open well chamber (73). Hopke et al. showed that isolated neutrophils could swarm and respond to micropatterned *Candida albicans* spots using the swarming assay. The authors further demonstrated that neutrophils kill and arrest the growth of *Candida albicans* over time by producing ROS and releasing NETs within the swarm (73) (**Figure 2C**). In conclusion, various studies have shown that pathogens play a crucial signaling role in modulating migratory and chemotactic abilities in neutrophils. Microfluidic devices are helpful to investigate the underlying mechanisms involved in neutrophil-pathogen interactions.

Microfluidic devices have also been designed and developed to use whole blood directly to investigate neutrophils and other immune cells in their most physiological environment. This concept bypasses the neutrophil isolation process from whole blood as it may lead to the pre-activation of neutrophils. In addition, these devices require the use of lower blood volume compared to other microfluidic models. For example, a study by Ellett et al. designed a microfluidic device with channels and mazes to measure different neutrophil motility patterns using whole blood samples. Briefly, the device consists of a single loading chamber and red blood cells filter channels at the periphery of the chamber. The RBC filter facilitates the confinement of the sample to the center of the device and concurrently allows migration of neutrophils to the assay field. It also prevents the entrance of other leukocytes that are larger and less deformable compared to human neutrophils. Ellett and colleagues showed measurement of spontaneous neutrophil motility in whole blood using this device (84).

Furthermore, a study by Otawara et al. also demonstrated the use of a microfluidic device to capture NETs released from neutrophils in whole blood following burn injury or sepsis (69). Briefly, the microfluidic device consists of two arrays of micro-posts arranged in series in a straight microchannel. The distance between adjacent micro-posts is designed and optimized to trap released chromatin fibers or NETs efficiently yet let blood cells flow through. To capture chromatin or NETs released, blood samples were flowed through the micro-post using a pump machine. As the blood samples flow-through the devices, chromatin fibers or NETs released are captured in the post array. The advantage of these devices is that it allows fast and non-invasive diagnosis of various innate immune-related diseases. The described devices have helped characterize neutrophil behavior *ex vivo*. However, they do not truly mimic the three-dimensional micro-environment and the extravasation process in neutrophils *in vivo.*

### Three-Dimensional Microfluidic Device to Investigate Neutrophil Behavior

Researchers have designed and developed microfluidic devices that allow the seeding of multiple cell populations to investigate their interactions in three-dimensional microenvironments. Neutrophils need to first interact with endothelial cells *via* extravasation in response to pathogen infection. As such, there have been microfluidic designs to study neutrophil-endothelial interactions (85, 86). These devices commonly include a collagen hydrogel that is coated with a monolayer of endothelial cells (**Figure 2D**); thus, neutrophil adhesion, extravasation, and migration through the hydrogel can be tracked and recorded using time-lapse microscopy. Multiple studies have recently demonstrated a microfluidic device that closely replicates *in vivo* physiology by incorporating relevant and critical components of three-dimensional structures to study neutrophil-immune responses (87–89). Incorporating these relevant and crucial components that mimic the *in vivo* microenvironment such as an endothelial vessel lumen in a three-dimensional microfluidic model is becoming more common and relevant (**Figure 2D**). These devices are designed by seeding collagen gel to mold a hollow structure that is then coated with endothelial cells to form a monolayer. Different strategies are involved in the fabrication and incorporation of flow into these devices, and they differ from one model design to another. Fabrication of this device is done using the viscous finger patterning technique to create a continuous lumen in hydrogels within microchannels (90). The viscous finger patterning approach was further developed into the LumeNEXT system (91).

The LumeNEXT platform relies on polymerizing a collagen hydrogel solution around a PDMS rod that is removed in order to generate a lumen. Barkal et al. (92) and Hind et al. (93) reported the application of this system to investigate the interaction of immune cells and live microbes and cell-cell interaction, respectively. Briefly, Barkal and colleagues described the interaction between neutrophils and fungi by monitoring and analyzing the migratory ability of neutrophils using a human organotypic lung 3D microfluidic device (92). The human bronchiole organotypic model comprises three cell-lined lumens within a 3D matrix of collagen and pulmonary fibroblasts. The center lumen and the two flanking lumens are lined with primary human bronchial epithelial cells and primary human lung microvascular cells respectively. This organotypic bronchiole model is set up to mimic *in vivo* function with an air-liquid interface; the seeded fibroblasts provide physiological support for endothelial and epithelial cells, forming physical barriers between the luminal and matrix spaces. A recent study by McMinn and colleagues reported a modular LumeNEXT system that allowed the user to disassemble the platform to retrieve cells from different locations of the hydrogel. The advantage of this system is that distinct neutrophil subpopulations can be sorted/collected based on their migratory ability. Furthermore, LumeNEXT has also been reported to monitor and investigate neutrophil-*Pseudomonas aeruginosa* interaction in real-time (**Figure 2D**). The authors showed that endothelium significantly increases the viability and migration of neutrophils towards live *P. aeruginosa* than neutrophils migrating through lumen alone (23).

## Neutrophil Interactions With Other Immune Cells

**Figure 3:**
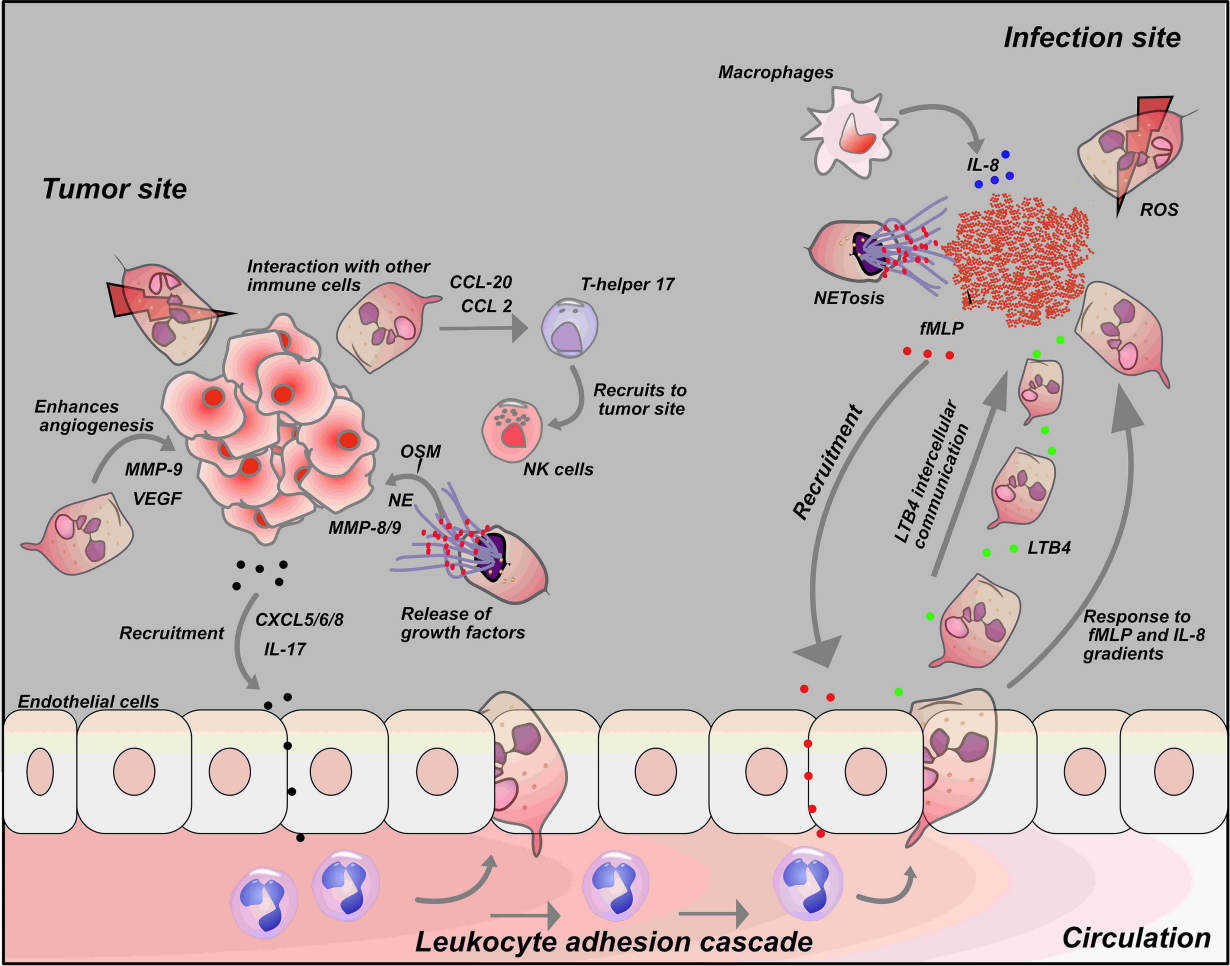
Neutrophil interaction with tumor cells and other immune cells: Interactions with tumor cells and other immune cells influences the neutrophil response. Tumor cells release chemokines such as CXCL-5, CXCL-6, CXCL-8 and IL-17 to recruit neutrophils to tumor cells. Secretion of chemokines such as CCL-20 and CCL-2 by neutrophils at tumor site activate T-helper 17 that in turn recruits anti-tumor immune cells like NK- cells to tumor cells. On the other hand, neutrophils can also release growth factors like NE, MMP-8/9 and VEGF at tumor sites that drives tumor metastasis and growth (Top left). At the infection site, fMLP, a known chemoattractant released by bacteria recruit neutrophils to the infection site. Neutrophils produce a secondary chemoattractant known as LTB_4_ that drives intercellular communication among neutrophils and recruit more neutrophil to the infection site in a process described as swarming. Macrophages also induce neutrophil migration by releasing a potent chemoattractant, IL-8 at the infection site (Top right).

During an inflammatory response, neutrophils must interact and communicate with other immune cells in a variety of ways that are crucial for the immune response. After transmigration, neutrophils must also navigate complex blood vessel networks and ECM to reach the infection site. To achieve this, neutrophils must migrate toward the chemokine gradient produced by other immune cells and pathogens at the site of infection (**Figure 3**). During an immune response, neutrophils signal to other migrating neutrophils. The interaction is through a signal amplification mechanism known as swarming. Neutrophil swarming has been described as an essential tissue response that is orchestrated to protect healthy tissue from unnecessary inflammation, limiting neutrophil migration to the pathogen-infected tissue (94). The role of neutrophil swarming during infection has been seen to be context dependent. It is essential for arresting the growth and spread of various pathogens (82, 83, 95–97).

Interestingly, it has been shown that response to swarming signals depends on LTB_4_ (98). It varies in magnitude depending on the pathogen type, thus indicating a role for the pathogen in modulating neutrophil function (73). In addition, swarming has been reported in a cell line model of neutrophils. A study by Babatunde et al. demonstrated that differentiated human leukemia- neutrophil-like cells (dHL-60) could exhibit swarming ability toward a cluster of zymosan particles using a swarming microfluidic device. The authors demonstrated that swarming in dHL-60 cells is comparable to primary neutrophils through quantitative but not qualitative data. The authors also showed that swarming in dHL-60 cells depends on the expression and secretion of LTB_4_ during coordinated migration toward the zymosan particles cluster (80) (**Table 4**).

**Table 4:** Summary of studies on neutrophil interaction with other immune cells.

Cell type	Model	Major finding	Ref.
**Neutrophil and other immune cells interaction**			
Neutrophil	*Ex vivo*: Microfluidic	Intracellular communication among neutrophils is driven by LTB_4_	(66)
			(73)
Monocytes	*Ex vivo*: Microfluidic	Monocytes promote neutrophil response in an LPS dependent manner	(86)
**Neutrophils-pathogen interaction**			
Fungus	*In vitro*	Established chemoattractant gradients primed neutrophils to inhibit fungi growth.	(76)
	*Ex vivo*: Microfluidic	Neutrophils exhibited an immune response to *A. fumigatus via* paracrine and autocrine signaling	(86)
Bacterium	*In vitro*	Neutrophils exhibited an immune response to bacteria compared to LPS	(74)
	*Ex vivo:* Microfluidic	The activation of endothelial cells by *P. aeruginosa* increased migration in neutrophils	(22)

Monocytes are also known to migrate from the blood vessels into the surrounding tissue. Once in the tissue, they differentiate into either macrophages or dendritic cells (99). Neutrophil-monocyte interaction has been reported using LumeNEXT. For example, a study demonstrated that monocytes significantly increase chemotactic response in neutrophils to *A. fumigatus* infection. The authors also showed that the observed increase in chemotactic response was macrophage inflammatory protein (MIP-1) dependent (93). Though few studies use microfluidic devices to study neutrophil-monocyte interactions, simple *in vitro* models, have also been used to demonstrate cellular crosstalk between monocytes and neutrophils. Zhang et al. show that macrophage-derived exosomes activate ROS production and NETosis in neutrophils (93).

The interaction of neutrophils and dendritic cells has not been studied using microfluidic platforms that mimic the *in vivo* microenvironment. However, simple *in vitro* approaches have been used to study the crosstalk between these cell types. For example, a study demonstrated that *Aspergillus fumigatus*-infected dendritic cells induce a chemotactic response in neutrophils by activating the IL-8 receptor (100). Other immune cells like T cells, mast cells, and natural killer cells (NK cells) have also been reported to interact with neutrophils. Pelletier et al. demonstrated that neutrophils induce chemotaxis in T-helper 17 cells by releasing CCL2 and CCL20 *in vitro* (101). In another report by Tamassia et al., the authors reported that neutrophil-secreted IL-23 induces naive CD4 T cells to differentiate into Th17 cells (102).

Neutrophil and Natural killer (NK) cells interactions have been shown to result in changes in the expression level of neutrophil receptors and survival. For example, a study showed that activated NK cells secrete factors and signals necessary for neutrophil survival and increase the expression of receptors including CD64, CD11b, and CD69 on neutrophils (103). In contrast, a study demonstrated that NK cells could induce apoptosis of neutrophils in a caspase-dependent manner (104). In addition, NK cells have also been reported to drive the apoptotic process in neutrophils following ROS-induced NK interaction (105). Neutrophil interactions with other immune cells like NK cells, dendritic cells, and macrophages play a crucial role in their immune responses. Overall, the study of neutrophil interactions with other immune cells is active field where microfluidic models could offer a versatile tool to decipher the cellular and molecular mechanisms underlying such processes.

## Microfluidic Devices to Investigate the Role of Neutrophils in Cancer

Tumor-associated Neutrophils (TANs) are neutrophils that have infiltrated into the tumor microenvironment. TANs are functionally classified as a tumor-suppressing N1 or tumor-promoting N2 phenotype although it is likely that there is a spectrum of phenotypes between N1 and N2. Each subpopulation of TANs has a distinct role in the tumor. N1 neutrophils have potent anti-tumor activity mainly due to their release of pro-inflammatory or immunostimulatory cytokines, such as interleukin (IL)-12, tumor necrosis factor (TNF)-α, CCL3, CXCL9, CXCL10 (32, 106). In contrast, N2 neutrophils have strong immunosuppressive and tumor-promoting activity, including stimulation of tumor angiogenesis, invasion and metastases *via* various factors (107). The role of neutrophils in cancer and the tumor microenvironment is multifactorial, and still unclear.

Few studies have demonstrated using a microfluidic device to investigate neutrophil behavior in the tumor microenvironment. For example, a recent study by Surendran et al. designed a microfluidic device to study the role of neutrophils in the tumor microenvironment. The 3D microfluidic device is known as the tumor immune microenvironment (TIME)-on-Chip device and serves both as a neutrophil migration and 3D tumor invasion platform. Briefly, a tumor spheroid was formed and embedded within the collagen matrix. The tumor spheroid setup was then hybrid-integrated with 3D bioprinting-enabled microfluidic channels. The TIME-on-Chip mimics the *in vivo* tumor microenvironment with key physiological components like extracellular collagen matrix compared to 2D models. The authors reported that the tumor spheroid recruited neutrophils by a chemotactic process and led to NETosis. However, the released NETs stimulated the invasion of the tumor cells into the surrounding collagen matrix, in a manner comparable to transforming growth factor-beta (TGF-β) and IL-8 effects on tumor cells. Furthermore, they reported that the tumor invasion was reversed by a drug that inhibits the NET formation pathway in neutrophils (108).

Another study by Chen and colleagues described and characterized a microfluidics-based, *in vitro* assay featuring 3D perfusable microvascular networks for studying tumor cell extravasation dynamics. The device is a self-organized human microvascular network formed by human umbilical vein endothelial cells (HUVECs) in fibrin gels, through which tumor cells can be perfused and extravasation events can be tracked *via* microscopy. The authors described a dynamic interaction between intravascular tumor cells and neutrophils at high spatiotemporal resolutions. They showed that neutrophil clusters were formed around the tumor cells. The clusters aggregation was chemotactically driven by neutrophil secreted IL-8 and tumor-derived CXCL1. However, the localization of the neutrophils around the tumor cells and secreted factors imply that it increases tumor extravasation potential by modulating the endothelial barrier (109).

Microfluidic devices could play an important role in improving our knowledge of how neutrophil related processes like ROS production and NETosis reduces NK and T cell tumor cytotoxicity and drives tumor progression (**Figure 3**). As such, a novel device has to be designed to allow the incorporation of these different immune cell populations to allow real-time monitoring and analyses of these interactions in a microenvironment that mimics the TME *in vivo.* Furthermore, the factors that drive neutrophil phenotype into pro-tumorigenic N2 neutrophils and anti-tumorigenic N1 cells remain unclear; microfluidic platforms could be useful in characterizing and identifying these factors.

## Microfluidic Device to Investigate the Neutrophil Role in COVID-19

Coronavirus disease 2019 (COVID-19) is a novel, viral-induced respiratory disease that in ∼15% of patients progresses to acute respiratory distress syndrome (ARDS) and its thought to be triggered by a cytokine storm (110). Neutrophils are present in many lung diseases associated with ARDS, including infections with influenza virus and COVID-19 (111). Increases in neutrophils has been identified as an indicator of severe respiratory symptoms and poor outcome in patients with COVID-19 (112–116). Elevated neutrophil numbers have also been reported in the nasopharyngeal epithelium of individuals infected with COVID-19 (117) as well as in the inferior lobe of the lung (118). Furthermore, plasma levels of neutrophil associated to factors such as resistin (RETN), lipocalin-2 (LCN2), and hepatocyte growth factor (HGF), were recently proposed as biomarkers for critical illness and mortality during COVID-19 (115).

Viral infection can also induce the release of NETs by neutrophils (119) NETs are known to immobilize and degrade pathogens such as bacteria, fungi, viruses, being a critical effector mechanism to contain infections (120). Several clinical and experimental studies have demonstrated an elevated level of NETs in individuals infected with COVID-19 (121, 122), and an increase in NETs in plasma is correlated with increased COVID-19 severity (123). NET formation in the lungs may have been triggered by direct contact of COVID-19 infected neutrophils. This has been shown *in vitro* as COVID-19 induced NET formation by neutrophils (123–125). In addition, the interaction between neutrophils and platelets has been implicated in the mechanism involved in increased NET levels during severe COVID-19 (126). The process of immunothrombosis can lead to blockage of hepatic micro-vessels causing hepatic cell death, thereby contributing to impaired functions of the lung. During severe COVID-19, the risk of immunothrombosis is increased by vasoconstriction driven by excessive cytokine release (127). In addition, a study by Wang and colleagues demonstrated at the transcriptional level the activation of several NETs-associated genes such as myeloperoxidase (MPO) and neutrophil elastase (NE) in individuals infected with COVID-19. The authors further hypothesized that the observed molecular changes could be as a result of negative regulation of anti-viral immune cells such as NK and T cells (128). This cascade of events associated with neutrophils triggered in COVID-19 infection undoubtedly contributes to the disease severity, thus there is a need to have a better understanding of the role of neutrophil in COVID-19.

There are limited studies using microfluidic devices to understand the role of neutrophils in COVID-19. Most studies to date are *in vitro* (123), *in vivo* using mouse models (129) and patients samples (129). However, microfluidics can provide a platform to help understand the neutrophil interactions with COVID-19 and how these interactions drive the pathophysiology of COVID-19 *ex vivo*. Reverse migrated neutrophils may become mechanically entrapped in the microvasculature of major organs such as lungs, thus contributing to its damage and failure as observed in COVID-19 (130). We postulate that the observed increased number of neutrophils during COVID-19 infection may be due to the tissue damage induced by the virus. Importantly, the presence of neutrophils in the lungs and nasopharyngeal epithelium during COVID-19 infection may be due to reverse migration of neutrophils back to the lungs from infection sites. If this is the case, microfluidic devices can provide a platform to study the interaction of COVID-19 and neutrophils *ex vivo* in a micro-environment that incorporates other immune cells such as endothelial cells, macrophages, NK and T cells in an environment that closely mimics the hepatic environment *in vivo*.

## Conclusion

Neutrophils are crucial to the body’s innate immune response as they are responsible for fighting against invading pathogens as well as cancer. During infection, neutrophils must process several inflammatory signals from their microenvironment in other to elicit their immune response. Several studies have tried to understand how neutrophils process and respond to these signals using various experimental systems that include *in vitro* and *in vivo* models.

Neutrophil phenotypes can exist as either pro-inflammatory, anti-tumor “N1” neutrophils or anti-inflammatory, pro-tumor “N2” neutrophils. Currently, it’s not clear if these different phenotypes of the neutrophil have an active role in reverse migration. It will be interesting to know if reverse migration specifically of N1 neutrophils can result in systemic inflammation or if reverse migration in the “N2” neutrophil phenotype could serve as a means of cancer immunotherapy. We speculate that increasing reverse migration in N2 neutrophil phenotype from TME and driving forward migration of N1 phenotype toward TME could limit tumor progression. There are limited studies demonstrating the use of microfluidic devices to understand the role of neutrophils in cancer. To understand how reverse migration in the N2 neutrophil phenotype could reduce tumor progression in the TME, we suggest that a novel microfluidic platform that allows the *in vitro* polarization of the N2 neutrophil phenotype towards the N1 neutrophil phenotype using suggested/known factors responsible for driving these phenotypes. Furthermore, this microfluidic platform should allow the monitoring and investigation of the interaction of neutrophils and tumor cells in single-cell resolution and in real-time in a microenvironment that mimics the TME *in vivo*.

Retrotaxis in neutrophils has been observed using both *in vitro* and *in vivo* models. It has been identified as a way to resolve inflammation, though it could also result in the spread of infection into the blood circulation. So far, *in vitro* and *in vivo* models have improved our understanding of the mechanisms involved in the neutrophil immune response. However, how these signals result in an effective neutrophil response following an infection remains unclear. Future studies should then focus on developing and designing devices that will include cell populations known to influence reverse migration in neutrophils, like macrophages. This will facilitate investigation of the factors driving the yet to be understood reverse migration of neutrophils in a microenvironment that closely recapitulates tissues *in vivo*. However, challenges remain as different immune cell populations need different culture periods, nutrients/media, and conditions *in vitro*, which poses additional hurdles to decipher the complex immune interactions during human disease. We suggest that different cell populations that are involved in the multicellular signaling cascades that drive reverse migration in neutrophils should be incorporated into the design of these microfluidic devices.

## Author Contributions

KB drafted the article. KB prepared the figures. JA, SK, AH, and DB provided critical feedback. All authors contributed to the article and approved the submitted version.

## Funding

This work supported by the University of Wisconsin Carbone Cancer Center, Cancer Center Support Grant NIH P30CA014520. This work was also supported by the National Institutes of Health NIH R01AI134749 to AH and DB, the National Institutes of Health NIH U24AI152177 to DB, and the Swiss National funding (P500PB_203002) to KB.

## Conflict of Interest

DB holds equity in Bellbrook Labs LLC, Tasso Inc., Salus Discovery LLC, Lynx Biosciences Inc., Stacks to the Future LLC, Turba LLC, Flambeau Diagnostics LLC, and Onexio Biosystems LLC. DB is also a consultant for Abbott Laboratories.

The remaining authors declare that the research was conducted in the absence of any commercial or financial relationships that could be construed as a potential conflict of interest.

## Publisher’s Note

All claims expressed in this article are solely those of the authors and do not necessarily represent those of their affiliated organizations, or those of the publisher, the editors and the reviewers. Any product that may be evaluated in this article, or claim that may be made by its manufacturer, is not guaranteed or endorsed by the publisher.
